# Exploring the role of L209 residue in the active site of NDM-1 a metallo-β-lactamase

**DOI:** 10.1371/journal.pone.0189686

**Published:** 2018-01-02

**Authors:** Francesca Marcoccia, Hanna-Kirsti S. Leiros, Massimiliano Aschi, Gianfranco Amicosante, Mariagrazia Perilli

**Affiliations:** 1 Dipartimento di Scienze Cliniche Applicate e Biotecnologiche, Università degli Studi dell’Aquila, L’Aquila, Italy; 2 The Norwegian Structural Biology Centre (NorStruct), Department of Chemistry, Faculty of Science and Technology, UiT The Arctic University of Norway, Tromsø, Norway; 3 Dipartimento di Scienze Fisiche e Chimiche, Università degli Studi dell’Aquila, L’Aquila, Italy; Russian Academy of Medical Sciences, RUSSIAN FEDERATION

## Abstract

**Background:**

New Delhi Metallo-β-Lactamase (NDM-1) is one of the most recent additions to the β-lactamases family. Since its discovery in 2009, NDM-1 producing Enterobacteriaceae have disseminated globally. With few effective antibiotics against NDM-1 producers, there is an urgent need to design new drug inhibitors through the help of structural and mechanistic information available from mutagenic studies.

**Results/Conclusions:**

In our study we focus the attention on the non-catalytic residue Leucine 209 by changing it into a Phenylalanine. The L209F laboratory variant of NDM-1 displays a drastic reduction of catalytic efficiency (due to low k_*cat*_ values) towards penicillins, cephalosporins and carbapenems. Thermofluor-based assay demonstrated that NDM-1 and L209F are stable to the temperature and the zinc content is the same in both enzymes as demonstrated by experiments with PAR in the presence of GdnHCL. Molecular Dynamics (MDs) simulations, carried out on NDM-1 and L209F both complexed and uncomplexed with Benzylpenicillin indicate that the point mutation produces a significant mechanical destabilization of the enzyme and also an increase of water content. These observations clearly show that the single mutation induces drastic changes in the enzyme properties which can be related to the observed different catalytic behavior.

## Introduction

Years of antibiotic therapies have promoted the development of antibiotic resistance in Gram-positive and Gram-negative bacteria [1]. Antibiotic resistance can arise from different mechanisms and the most common is promoted by β-lactamases. This class of enzymes can be distinguished into serine-β-lactamases (molecular classes A, C and D) and metallo-β-lactamases (MBLs) [2]. Unlike serine-β-lactamases, MBLs show a different catalytic mechanism, where they require zinc ions to catalyze the hydrolysis of β-lactams. According to their sequence and alignments, MBLs can be further divided into three subclasses: B1, B2 and B3 [3]. The enzymes included into these subclasses shared a low degree of sequence similarity and some differences in secondary structures elements. Inside subclass B1, a new metallo-β-lactamase named New-Delhi metallo-β-lactamase (NDM-1) has been discovered [4]. The rapid spread all over the world of this enzyme is due to [5] its genetic localization on complex mobile elements, which increases the dissemination among different strains of Gram-negative bacteria, and the absence of useful inhibitors for the enzyme, preventing the possibility to fight the infections caused by NDM-producing bacteria [6]. NDM-1 displays 32% of sequence identity to the most common MBLs, IMP-1 and VIM-2 [7]. In these enzymes substrate catalysis is promoted by two zinc ions which are coordinated by H120, H122, H189 (Zn1, site 1) and D124, C208 and H250 (Zn2, site 2) (NDM-1 numbering). In NDM-1, the Zn1 is tetracoordinated by the imidazole groups of three histidine residues and one water molecule, whereas Zn2 is pentacoordinated by H250, D124, C208 and one water molecule. Two important loops surround the active site: Loop 3 and Loop 10, which have a greater flexibility and facilitate the entrance of the substrates. Loop 3 is a short loop present in most B1 MBLs and it includes residues 67–73; loop 10 is a long loop and it goes from residue 210 to 230 [8].

**Fig 1:**
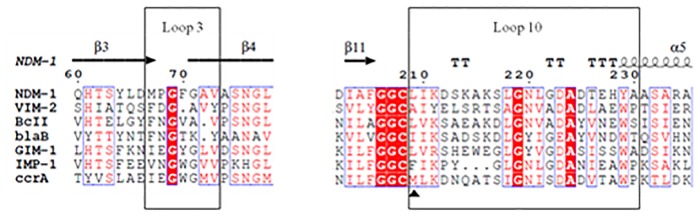
Sequence alignment of NDM-1 with the sequence of some B1 MBLs. In the top is shown the secondary structure annotation of NDM-1 (PDB-ID 3Q6X). Arrows indicates β-strands, TTT and TT strict α and β-turn respectively; spirals indicate α-helices. Red letters indicate similar residue; red background indicate the identical residues, and blue boxes indicate conserved position. Aligned sequence was made using EsPrit (v.3.0).

In the present study, we have focused the attention on residue L209 located in the Loop 10 of NDM-1, as shows by Fig 1. L209 interacts with the conserved residue Y229, by forming a hydrogen bond [9]. Moreover, hydrophilic and hydrophobic network created by L209 and the neighboring residues seems to stabilize Loop 10. In addition, L209 is the following residue after C208, which is a Zn2 binding ligand and also the residue that belong to the GGC stretch (G206-G207-C208). The GGC region has a remarkably conformational freedom, mainly due to the presence of two residues of glycine. Its flexibility allows alternative conformation of C208 promoting the binding of different substrates to the Zn2 ion [10]. Site-directed mutagenesis was used to replace leucine 209 to phenylalanine. This substitution was chosen on the basis of the alignment of NDM-1 with the most common B1 MBLs (Fig 1). Leucine at this position is present in NDM-1, BCII and GIM-1 enzymes but is not a conserved residue. In order to inspect the role of L209, both L209F variant and NDM-1 were investigated under various aspects: thermal stability, kinetic features, molecular dynamics (MD) and antimicrobial susceptibilities.

## Methods

### Strains and vectors

*E*. *coli* NovaBlue (*endA1 hsdR17*(r_K12_^−^ m_K12_^+^) *supE44 thi-1 recA1 gyrA96 relA1 lac* F′[*proA*^*+*^*B*^*+*^ *lacI*^*q*^*Z*Δ*M15*∷Tn*10*] (Tet^R^)) was used for the initial cloning as a nonexpression host. *E*. *coli* BL21(DE3)codon-plus (*E*. *coliB F-ompT hsdS(rB-mB-)dcm+Te trgalλ(DE3)endAHte [argU proLCamr*]) was used for overexpression of the mutant. The pET-24a(+) vector was used for cloning experiments and pFM-NDM-1 recombinant plasmid [11] was used as template for site-directed mutagenesis.

### Antibiotics

Nitrocefin was kindly provided by Shariar Mobashery (Notre Dame University, South Bend, IN, USA). The chemical composition of nitrocefin is available on Sigma-Aldrich company. Meropenem was from AstraZeneca (Milan, Italy). Imipenem from Merck Sharp & Dohme (Rome, Italy). Biapenem was from Cyanamid (Catania, Italy). Loracarbef was from Eli Lilly and Co. (Indianapolis, IN, USA). The other antimicrobial agents used in this study were purchased from Sigma-Aldrich (Milan, Italy). The molar extinction coefficients and wavelengths used in the assay were: imipenem Δε_300_ = -9000 M^-1^ cm^-1^, meropenem Δε_297_ = -6500 M^-1^ cm^-1^, biapenem Δε_293_ = -7290 M^-1^ cm^-1^, loracarbef Δε_260_ = -11300 M^-1^ cm^-1^, cefazolin Δε_260_ = -7400 M^-1^ cm^-1^, carbenicillin Δε_235_ = -780 M^-1^ cm^-1^, benzylpenicillin Δε_235_ = -775 M^-1^ cm^-1^ and nitrocefin Δε_482_ = +15000 M^-1^ cm^-1^.

### Site-directed mutagenesis, cloning, enzyme expression and purification

The L209F mutant was generated by site directed mutagenesis using the overlap extension method [12]. Primers NDM_for 5′-GGGGG**CATATG**GGTGAAATCCGCCCGA and NDM_rev 5′- GGGGG**CTCGAG**TCAGCGCAGCTTGTCGGC (in bold are indicated the restriction site sequences) were used to amplify the entire gene. Primers L209F_for 5′-GGCTGCTTCATCAAGGAC and L209F_rev 5′-GTCCTTGATGAAGCAGCC (the mutated sequence is underlined) were used for site-directed mutagenesis. The mutated gene was cloned into pET-24a(+) digested with *Nde*I and *Xho*I restriction enzymes. The procedures for over-expression and purification of L209F were the same used for NDM-1 as previously reported [11].

### Determination of kinetic parameters

Steady-state kinetic experiments were performed following the hydrolysis of the β-lactams at 25°C in Hepes 20 mM pH 7.0 + 20 μM of ZnCl_2_. The data were collected with a Perkin-Elmer Lambda 25 spectrophotometer (Perkin-Elmer Italia, Monza, Italy). Kinetic parameters were determined under initial-rate conditions using GraphPad Prism6 software to generate Michaelis-Menten curves or by analyzing the complete hydrolysis time courses [13]. Each kinetic value represents the mean of the results of three different measurements; the error rate was below 5%. To calculate k_cat_ values we used the theoretical molecular weight of 25605.87 Da, assuming cleavage of signal peptide at position of residue G29 (G24 according to BBL standard numbering) as predicted from SignalP 4.0 [14].

### Antimicrobial susceptibility

To perform MIC experiments the *bla*_NDM-1/L209F_ and *bla*_NDM-1_ with (SP) were cloned into pBC-SK(+) vector and recombinant plasmids were inserted by transformation in *E*. *coli* XL-1 competent cells. The phenotypic profile has been characterized by a microdilution method using a bacterial inoculum of 5X10^5^ CFU/ml according to Clinical and Laboratory Standards Institute (CLSI) performance standards [15].

### Thermofluour assay

The thermal stability of wild type NDM-1 and mutant L209F was determined using a fluorescence based thermal stability assay in a MJ minicycler (Bio-Rad). The assay volume used was 25 μl, which included 2 μM of enzyme, 25x SYPRO Orange solution (diluted from the 5000x stock provided by Sigma-Aldrich, US), and 20 mM Hepes pH 7. Thermostability was tested in absence and presence of 100μM ZnCl_2_. The temperature gradient was from 10 to 70°C with an increasing of 1°C per minute. The melting temperatures (T_m_s) were determined to be the inflection point of the melting transition found from the first derivative. All experiments were performed in triplicate.

### Computational details

All the simulations were carried out using the Gromacs package, version 4.5.5 [16] with the Gromos force-field (gromos54a7) [17]. The charges of the substrate (benzylpenicillin) and active site were calculated using standard fitting procedures with the program Gaussian 09 [18, 19]. For the simulations, the enzyme was inserted in a cubic box filled with 7965 molecules of water described with the Simple Point Charge (spc) model [20]. The dimension of the box was adjusted for correctly reproducing the density of the system at 298 K and 1.0 bar of pressure as previously described [21]. The productive simulations were then performed in the NVT ensemble with a time step of 2.0 fs, the temperature was kept constant using the velocity rescaling procedure [22]. The LINCS algorithm was employed to constraint all bond lengths [23]. Long-range electrostatic interactions were computed by the Particle Mesh Ewald method with 34 wave vectors in each dimension and a fourth-order cubic interpolation, and a cut-off of 1.1 nm was used [24]. The productive simulations wereextended for different times, namely: 80 ns for uncomplexed NDM-1 and L209F and 100 ns for the complexes NDM-1/benzylpenicillin and L209F/benzylpenicillin.

Before running the productive simulations the systems were prepared as follows: after an initial energy minimization, the system was gradually heated from 50 to 250 K using short (20 ps) MD simulations. Finally, a further pre-equilibration of the system, arrived at 298K, was carried out by running 5.0 ns of MD simulation in all the systems. Note that all these equilibration trajectories were disregarded by the following analysis. All the analysis were performed either using the tools contained in the Gromacs package or adopting simple in-house made routines.

### Determination of Zinc content using PAR under denaturing conditions

The determination of Zinc content of the enzymes was determined under denaturing conditions using the colorimetric metal chelator 4- (2- pyridylazo) resorcinol (PAR) as described by Fast et al [25].

### Modelling of the simulated systems

The starting coordinates of the four simulations were obtained by modifying the crystal structure of NDM-1 coordinated to hydrolyzed benzylpenicillin (hereafter termed BzP) taken from Protein Data Bank (PDB accession number: 4eyf [26]. Based on previous experimental and computational studies [7, 27–30] the modifications were performed adopting the following strategy: a) for the simulations of uncomplexed NDM-1 we introduced the hydroxide ion, acting as the nucleophile, shared between the two zinc ions both characterized by a tetrahedral coordination; b) for the simulations of complexed NDM-1 we bound the BzP carbonyl group to the Zn1 and the BzP amide nitrogen and carboxylate group to Zn2; c) for the simulations of L209F the above protocol was repeated with the mutation performed with the program Molden [31]. It is important to remark that the zinc ions were considered as bound to the ligands and hence the corresponding bonds were treated as covalent bonds along the simulation.

### Collective analysis of the protein framework

Internal mobility of protein framework was carried out using a standard method based on the C-alpha Root Mean Square Fluctuation analysis (RMSF). In order to quantify the overall backbone fluctuation we performed Essential Dynamics (ED) analysis [32]. At this purpose, for each system, we have first constructed and then diagonalized the backbone covariance matrix (Eq 1)

C=⟨(x-⟨x⟩)∙(x-⟨x⟩)T⟩ (1)

where x are the coordinates of backbone atoms and <x> is the average value.

The overall enzyme fluctuation has been then quantified through the trace of the matrix (1).

An estimation of the whole protein geometry and shape, not straightforward in the case of dynamical systems, has been performed through as below described [33, 34]. For each simulation, and at each frame, we constructed the 3x3 covariance matrix ($\tilde{M}$) (Eq 2).

$$\tilde{M}\;=\;\frac{1}{N}\sum_{i\;=\;0}^{n}\left(x_{i}-\langle x\rangle )∙(x_{i}-\langle x\rangle \right)T$$ (2)

where N is the total number of atoms of the protein and ‹x› is the vector whose components are the average components of all the atomic position.

Diagonalization of the above matrix (Eq 2) produces three eigenvectors representing the three axes (*a*_i_) of the instantaneous ellipsoid describing the protein with values given by *a*_*i*_ = 2√*l*_*i*_ where *l*_*1*_, *l*_*2*_ and *l*_*3*_ are the associated eigenvalues of matrix (Eq 2). The protein volume at each particular frame has been then approximated by the volume of the associated ellipsoid. Moreover, by simply evaluating the number of water molecules contained within the ellipsoid we have also evaluated the instantaneous internal hydration.

## Results and discussion

NDM-1 is a monomeric enzyme of 270 aa including a signal peptide of 28 aa. Compared with other MBLs it shares the characteristic αβ/βα fold structure and the same catalytic mechanism. Different to other MBLs, the NDM family does not include a large cluster of different variants most likely due to the fact that it arises recently from the “antibiotic resistance world”. Actually, since today, only 18 NDM-1 variants were discovered and differently to other MBLs, each variant shows a single or a double mutation respect to the prototype NDM-1. The alignment in Fig 1 shows, for Loop 3 and Loop 10, the conserved residues (red background) among different subclasses B1 MBLs and pointed out a low similarity between NDM-1 and other MBLs. As indicated in Fig 1, position 209 is conserved in terms of similar amino acid features (red letters), and the only one residue that shows different property, in terms of aromatic side chain, is the phenylalanine in IMP-1. In order to understand the role of a hydrophobic residue at position 209, leucine was replaced by phenylalanine in NDM-1 enzyme. Considering that, NDM-1 is anchored to the membrane when it is produced with its native signal peptide [35], the L209F mutant was produced and purified as previously reported for NDM-1 [11]. At the end of the purification, 2 mg of pure L209F enzyme (>95%) was obtained from 400 ml of cell culture.

Interesting results were obtained comparing K_m_, k_cat_ and k_cat_/K_m_ values of L209F with that obtained for NDM-1 [11]. The L209F enzyme displays a drastic reduction in the catalytic efficiency towards most substrates tested (Table 1). In particular, L209F showed a severe decrease of the k_cat_ values, even if the K_m_ values for all substrates remains similar to those calculated for NDM-1. With regards to carbapenems (imipenem, meropenem and biapenem) and cephalosporins (loracarbef and cefazolin) L209F showed a reduction ranging from 70 to 100 fold in the k_cat_/K_m_ values. An exception is represented by penicillins (benzylpenicillin and carbenicillin), for which the K_m_ values are lower at 7 and 20 μM, respectively. In this way, the L209F has lower K_m_ than the NDM-1 by 15 and 35 times for carbenicillin and benzylpenicillin respectively. However, the enhanced ability of L209F to interact with penicillins is not enough to enhance the enzymatic turnover (k_cat_). In fact, the catalytic efficiencies remain low but are only 3 and 5 -fold less than the NDM-1 enzyme.

**Table 1:** Kinetic parameters of NDM-1 and L209F enzymes.

Substrates	NDM-1			L209F		
	K_m_ (μM)	k_cat_ (s^-1^)	k_cat_/K_m_ (μM^-1^ s^-1^)	K_m_ (μM)	k_cat_ (s^-1^)	k_cat_/K_m_ (μM^-1^ s^-1^)
Imipenem	35±1	64	1.83	33±5	0.55	0.017
Meropenem	80±2	75	0.94	50±1	0.86	0.017
Biapenem	120±4	30	0.25	109±4	0.52	0.0047
Cefazolin	20±1	42	2.1	16±2	0.34	0.021
Loracarbef	40±2	42	1.05	20±3	0.78	0.039
Benzylpenicillin	250±10	105	0.42	7±0,5	1.00	0.140
Carbenicillin	285±5	108	0.38	20±3	1.40	0.070

**Fig 2:**
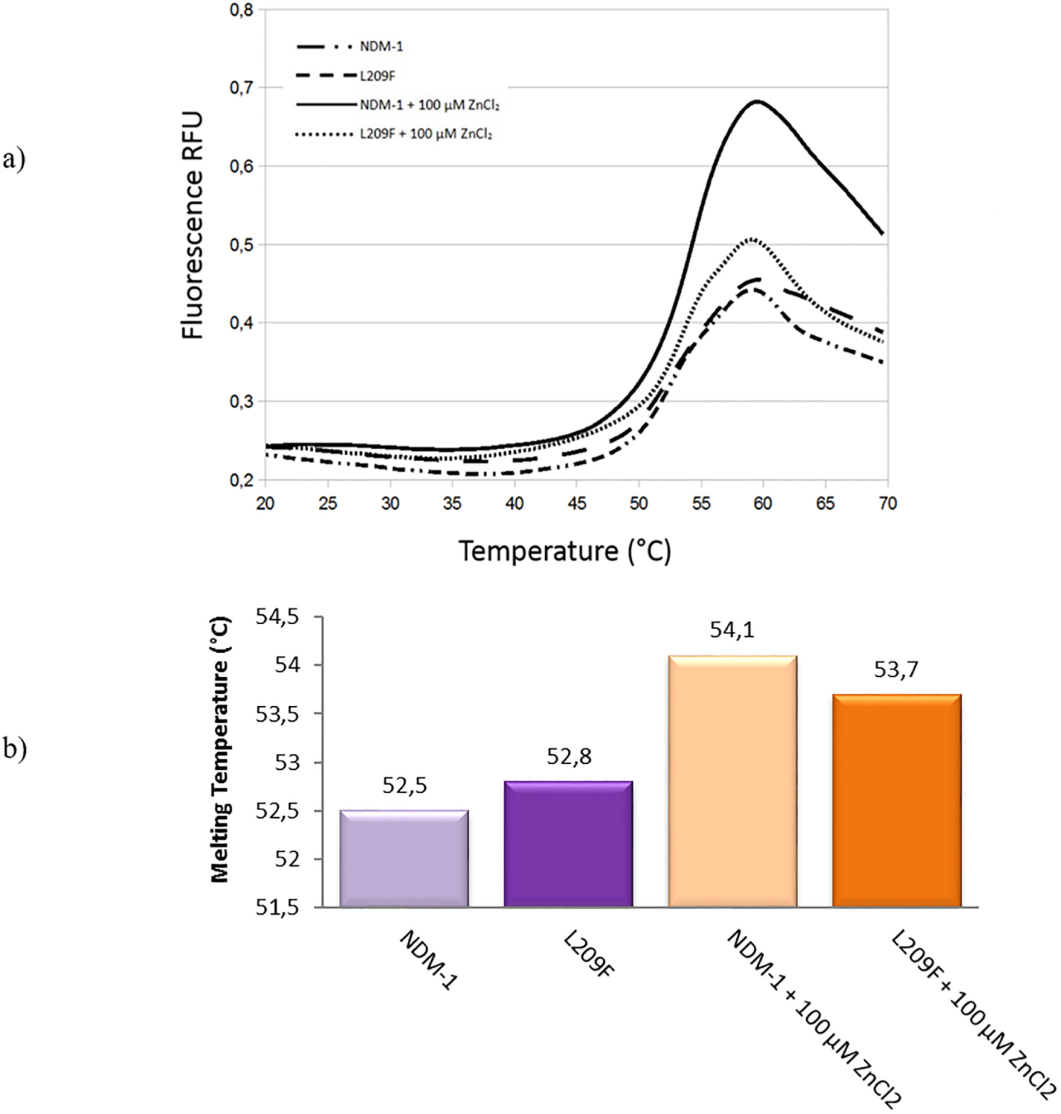
Thermofluor experiments of NDM-1 and L209F enzymes. Panel (a), melting curves from thermofluor experiments of NDM-1 and L209F enzymes with and without 100 μM ZnCl_2_. Panel (b), the melting temperatures are shown in °C.

The stability of the enzymes (NDM-1 and L209) was investigated using the thermofluor-based assay. As pointed out from the melting curves (Fig 2a), both the NDM-1 and L209F show roughly the same melting temperature. In fact, the differences between the enzymes are not more than 0.3–0.4°C ± 0.1 (Fig 2b), so we can assess that the mutant remains stable as NDM-1, and that the stability cannot explain the differences observed in the catalytic activities. We performed the experiment also in different buffer composition and pH, with and without 100 μM ZnCl_2_, NaCl and glycerol, but no diversity or difference between NDM-1 and mutant was encountered.

Interestingly, kinetic data were confirmed by antimicrobial susceptibility test (Table 2). When compared with *E*. *coli* XL-1/pBC/NDM-1, a drastic reduction of MIC values were observed for *E*. *coli* XL-1/pBC/L209F versus all β-lactams tested. Concerning carbapenems and cefepime, the *E*. *coli* XL-1/pBC/L209F have the same MIC values of *E*. *coli* XL-1/pBC-SK.

**Table 2:** Antimicrobial susceptibility test.

β-Lactams	*E*. *coli* BL21Codonplus DE3/pET24	*E*. *coli* BL21Codonplus DE3/pET24/NDM-1*	*E*. *coli* BL21Codonplus DE3/pET24/L209F*
Imipenem	0.5	>64	0.25
Meropenem	0.5	>64	<0.0625
Biapenem	<0.0625	16	<0.0625
Cefazolin	<0.0625	256	4
Cefotaxime	<0,0625	128	1
Ceftazidime	<0.0625	256	16
Cefoxitin	0.125	8	8
Cefepime	<0.0625	32	<0.0625
Loracarbef	<0.0625	128	16
Cefaclor	0.5	64	16
Moxalactam	<0.125	64	0.25
Benzylpenicillin	0.5	512	64
Carbenicillin	0.5	512	64

*Both blaNDM-1 and blaL209F are with signal peptide

To explain the low k_cat_ values and the low MIC obtained for L209F the total zinc content was determined by a spectrophotometric assay of colorimetric reagent 4-(2-pyridylazo) resorcinol. The measured metal content was 1.9 Zn(II)/NDM-1 and 1.82 Zn(II)/L209F. This suggest that the replacement of L209F does not alter the metal binding to the C208 site. Since, the zinc content values for both enzymes is similar we have presumed that the low k_cat_ values obtained for L209F enzyme do not depend on zinc content.

Molecular dynamics (MD) simulations were performed with the precise aim of observing structural-dynamical differences induced both by the introduction of the substrate and by the mutation. At this purpose we have performed simulations of NDM-1 and L209F uncomplexed and of NDM-1 and L209F complexed with benzylpenicillin (hereafter termed as BzP).

**Fig 3:**
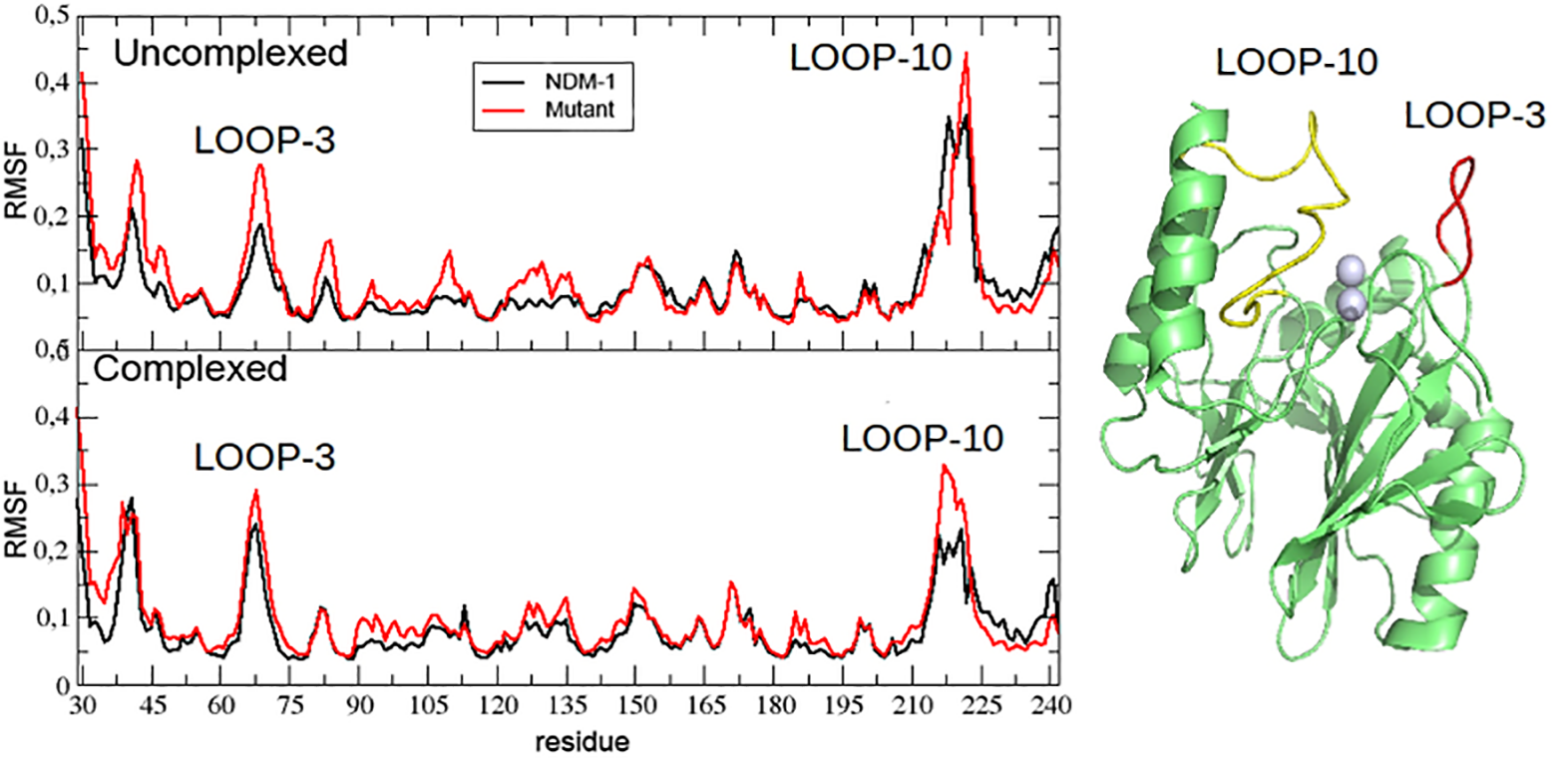
Root Mean Square Fluctuation (RMSF) of the investigated systems. The RMSF is expressed in nm^2^; in the inset Loop 3, residues 67–73 (red) and Loop 10, residues 210–230, (yellow) are schematically highlight.

A preliminary analysis of the enzyme internal mobility was based on the C-alpha Root Mean Square Fluctuation (RMSF) shown in the Fig 3. In all the four systems (complexed and uncomplexed NDM-1 and L209F) most of the fluctuation is concentrated over Loop 3 (M67-G73), Loop 10 (I210-A230) and over the loops next to the N-terminus and C-terminus. As suggested by Fig 3, we noticed that NDM-1 is, within the estimated error, the most rigid structure in terms of fluctuation and that the presence of BzP produces only a slight, although significant, increase of the internal flexibility. On the other hand, the fluctuation in L209F is systematically higher than in NDM-1, but not particularly affected by the presence BzP.

Further analyses have been also performed for revealing structural differences among the investigated systems. In particular, the number of internal contacts and the degree of hydration of the enzyme interior have been estimated as reported in the Computational details. All these results are collected in Table 3. First of all, we observe that the repertoire of all the monitored short-distance interactions turns out to be essentially unchanged, at least within the error, indicating that the all investigated systems maintain their stability and do not undergo any misfolding transition, at least within the simulated times. Secondly it is important to remark that L209F, both complexed and uncomplexed, shows significantly enhanced volume and backbone fluctuation probably induced by the sharp increase of water content, if compared to NDM-1.

**Table 3:** Effect of the solvent in the four systems NDM-1 and L209F and direct measure of the extent backbone overall fluctuation in complexed and uncomplexed system.

System	Average number of H bonds^a^^,^^b^	Average number of contacts ^b^^,^^c^	Average volume (nm^3^) and related fluctuation (nm^3^)	Water concentration and related fluctuation (molecules/nm^3^)	Backbone overall fluctuation and estimated maximum error (nm2)^d^
NDM-1 uncomplexed	184±6	1042±17	21.8±0.2	48.6±0.2	8.7±0.2
NDM-1 complexed	186±7	1047±16	21.7±0.2	46.8±0.3	9.0±0.2
L209F uncomplexed	179±7	1043±17	22.7±0.3	59.5±0.4	9.5±0.3
L209F complex	181±6	1045±15	22.4±0.3	54.9±0.4	9.6±0.3

^a^ We have utilized the standard criterium of the Groamcs tools for the considering as formed a given H-bond or contact.

^b^ Uncertainty is reported as maximum error evaluated considering two halves of the trajectory used for calculationg the average values.

^c^ We have considered as contacts the presence of inter-atomic distance lower than 0.35 nm.

^d^ The backbone fluctuation was calculated as the average between the traces of the diagonalized matrix (1) obtained using two halves of the trajectiry. The maximum error was calculated as the maximum error considering two halves of the trajectory.

It is important to note that, rather unexpectedly, the analysis of the water content in the active site of complexed NDM-1 (4±3) and L209F (3±3) does not reveal significant differences hence suggesting that the experimentally observed differences in the reactivity, in the present case, cannot be simply reduced to single observables and probably rely on very complicated non-local (enzyme and solvent) effects.

**Fig 4:**
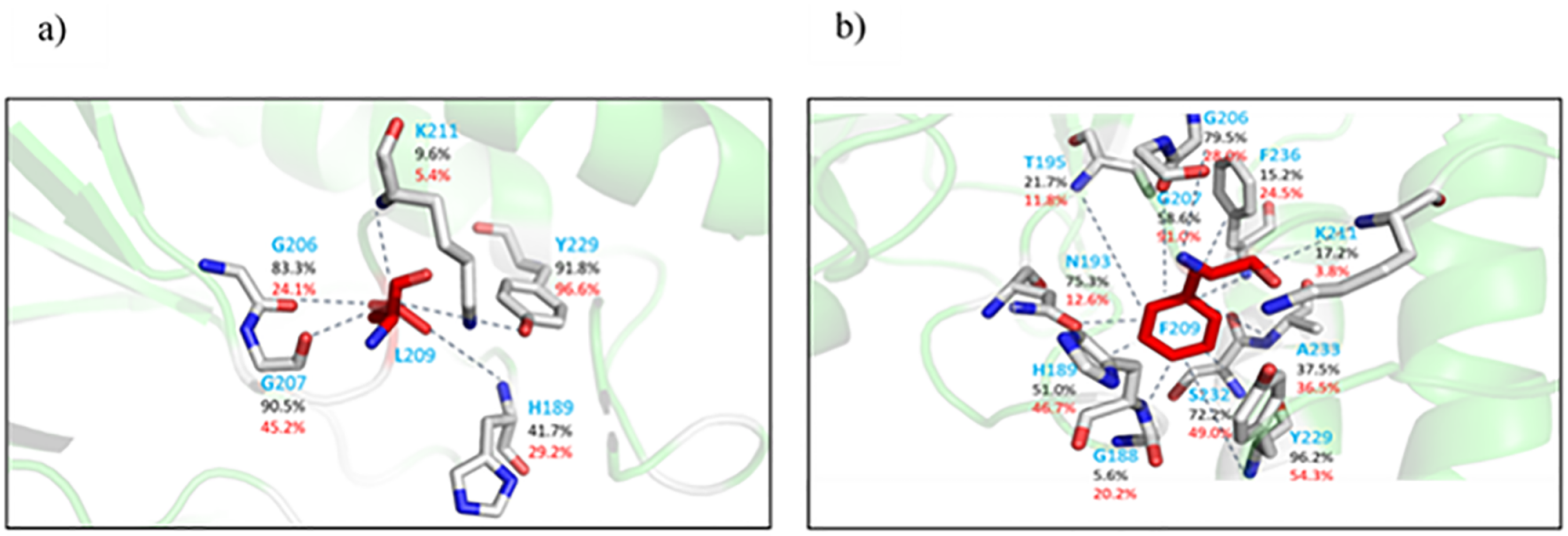
Network of contacts among L209 (a) and F209 (b) and neighbor residues. (a) percentage of occurrence of short-distance contacts in the case of free NDM-1 (black numbers) and complexed NDM-1 (red numbers). (b) percentage of occurrence of short-distance contacts in the case of free L209F (black numbers) and complexed L209F (red numbers).

As a final analysis we searched for a correlation between the observed differences and the presence of the mutation in the active site. This has been performed by monitoring the different contacts experienced by the residues in the position 209. In Fig 4(a) and 4(b) we schematically report the stability, expressed in terms of percentage of occurrence along the trajectories, of the non-bonding contacts, including H-bonds, between the mutated residue and the local environment including water molecules possibly present. The leucine 209 (Fig 4a) forms a number of extremely stable contacts. On the other hand, phenylalanine in the same position (Fig 4b) appears as interacting with a higher number of residues but with a reduced stability. This result indicates that mutation in position 209 actually produces a significant difference in the local interactions in the active site, not in disagreement with the sharp variations previously reported.

In the present study we have focused the attention on residue L209 located in the “second sphere residues” outside the enzyme active site. The “first sphere residues” includes residues that coordinate the zinc ions. In NDM-1 position 209 is occupied by a leucine residue that is able to interact through hydrogen bond with Y229 that plays an important role in controlling the stability and flexibility of Loop 10 [9]. Leucine at this position is present in NDM-1, BCII and GIM-1 enzymes but is not a conserved residue. Our results show that also a mutation in a residue of the second sphere of NDM-1 could strongly influence the catalysis. Studies reported in literature stated that not only the first sphere residues highly affect the activity of MBLs but also the second sphere residues influence negatively o positively the substrate hydrolysis [36]. This is the case of BcII enzyme, subclass B1 metallo-β-lactamase. As reported by Tomatis et al., some BcII variants that contains mutations outside of active site enhance catalytic efficiency towards β-lactams [36]. In fact, the G262S BcII mutant have evolved its hydrolytic profile respect to BcII enzyme conferring higher resistance levels of antibiotics. Differently, in our mutant, the F209 drastically reduce the catalytic efficiency towards all β-lactams tested. The F209 perturbs the interaction with neighboring residues promoting more general variations in the enzyme fluctuation pattern and geometrical features.

## Conclusion

In conclusion, computational results obtained in this study can be concisely summarized in a few key points: a) both NDM-1 and L209F represent stable species with respect conformational transitions because of the abundant network of internal contacts; b) both NMD-1 and L209F are characterized by dynamical-mechanical features scarcely affected by the presence of the substrate (BzP); c) the mutation, shown to drastically alter the local contacts of residue in position 209 produces also long-range sharp variations and in particular: (i) increase in the overall enzyme fluctuation (i.e. reduction of enzyme mechanical stability), (ii) increase of enzyme dimension and (iii) increase in the enzyme water content. All the emerged structural-mechanical differences, although not explicitly because of the high complexity of the systems, can be implicitly related to the catalytic differences observed in the experiments.
